# Meta-Analysis for the Prediction of Mortality Rates in a Pediatric Intensive Care Unit Using Different Scores: PRISM-III/IV, PIM-3, and PELOD-2

**DOI:** 10.3389/fped.2021.712276

**Published:** 2021-08-24

**Authors:** Yaping Shen, Juan Jiang

**Affiliations:** ^1^Department of Pediatrics, Shengzhou People's Hospital, the First Affiliated Hospital of Zhejiang University Shengzhou Branch, Shaoxing, China; ^2^NICU, Ningbo Women and Children's Hospital, Ningbo, China

**Keywords:** meta-analysis, pediatric intensive care unit, Pediatric risk of mortality, Pediatric index of mortality, Pediatric logistic organ dysfunction-2

## Abstract

**Introduction:** The risk of mortality is higher in pediatric intensive care units (PICU). To prevent mortality in critically ill infants, optimal clinical management and risk stratification are required.

**Aims and Objectives:** To assess the accuracy of PELOD-2, PIM-3, and PRISM-III/IV scores to predict outcomes in pediatric patients.

**Results:** A total of 29 studies were included for quantitative synthesis in meta-analysis. PRISM-III/IV scoring showed pooled sensitivity of 0.78; 95% CI: 0.72–0.83 and pooled specificity of 0.75; 95% CI: 0.68–0.81 with 84% discrimination performance (SROC 0.84, 95% CI: 0.80–0.87). In the case of PIM-3, pooled sensivity 0.75; 95% CI 0.71–0.79 and pooled specificity 0.76; 95% CI 0.73–0.79 were observed with good discrimination power (SROC, 0.82, 95% CI 0.78–0.85). PELOD-2 scoring system had pooled sensitivity of 0.78 (95% CI: 0.71–0.83) and combined specificity of 0.75 (95% CI: 0.68–0.81), as well as good discriminating ability (SROC 0.83, 95% CI: 0.80–0.86) for mortality prediction in PICU patients.

**Conclusion:** PRISM-III/IV, PIM-3, and PELOD-2 had good performance for mortality prediction in PICU but with low to moderate certainty of evidence. More well-designed studies are needed for the validation of the study results.

## Introduction

The main aim of the pediatric intensive care unit (PICU) is to decrease mortality in infants by both monitoring and treating critically ill patients who are considered at risk of dying. To provide the better quality of care with available resources and optimal management of such patients, a suitable management plan and prioritization of resource utility after the identification of “at-risk” patients are needed (1). In China, mortality rates associated with PICU admission are approximately two or three times higher than in America and Europe (2). It is, therefore, essential to identify predictors and determinants of death in PICU for the risk stratification and optimal management of such patients. Death prediction scores have been constantly explored by critical care health care providers since the establishment of PICU.

The scoring system aims to predict the outcome during treatment and to provide a better quality of care with available resources. Many mortality prediction scoring systems are being used for predicting outcomes in PICU patients. Although it is a complicated process to assess the individual patient outcome precisely, there have been efforts to develop and validate models for prediction accuracy of outcomes, such as Pediatric Risk of Mortality (PRISM) III/IV, Pediatric index of mortality (PIM-3), and Pediatric Logistic Organ Dysfunction-2 (PELOD-2). However, their predictive accuracy varied significantly in different populations worldwide (3–5).

The PIM was developed from data collected from PICUs in three prospective studies, from 1988 to 1995, and a cohort study, conducted from 1994 to 1997 by Shann et al. (6). PIM constructs a simple 10-variable model that is assessed at the time of admission to the PICU. Apart from the prediction of morality, this model also helps in the assessment of medical care quality and employment of resources. The revised version of the PIM study (PIM-3) has better calibration and discrimination capability than the previous model, PIM-2, reported in 2013 (7, 8).

PRISM score is another widely used model that was developed using data collected from PICUs in the United States. PRISM was later updated to PRISM-III and PRISM-IV with better calibration and discrimination efficiency (9) and is used to predict the risk of mortality during admission at PICU.

Finally, PELOD-2, another mortality prediction model developed in 2013, was also validated with excellent calibration and discrimination efficiency (AUC 0.934, calibration *p* = 0.317) (10).

Several recent studies have evaluated various prediction models to predict outcomes in PICU patients but have shown inconsistent findings, such as underestimation or overestimation of mortality prediction, poor discriminatory power, and absence of reporting of calibration statistics. (4, 11) As of today, there is no pooled evidence on the accuracy of these scores for PICU patients. The main goal of the current study is to conduct a systematic review and meta-analysis to evaluate the predictive accuracy of PRISM-III/IV, PIM-3, and PELOD-2 scores to predict mortality in pediatric patients in the PICU.

## Materials and Methods

**Study Design**: Systematic review and meta-analysis

**Ethical Clearance**: Not Required.

### Search Strategy

The present meta-analysis was conducted according to the reporting guidelines suggested in the PRISMA 2020 and Cochrane library. Search engines and electronic databases, such as Google Scholar, PubMed, and CENTRAL (Cochrane Central Register of Controlled Trials) were used to retrieve English language papers published up to May 2021. Free text words and medical subject heading (MeSH) terms were used, and the reference lists of potentially eligible studies and relevant review articles on a similar topic were scanned for additional possible studies. The following search key words were used: (((“pediatrics”[All Fields] OR “pediatrics”[MeSH Terms] OR “pediatrics”[All Fields] OR “pediatric”[All Fields] OR “pediatric”[All Fields]) AND (“pediatric Risk of Mortality” [All Fields] (“prism”[All Fields] OR “prism s”[All Fields] OR “prisms”[All Fields])) OR “Pediatric Logistic Organ Dysfunction-2” OR “PELOD”[All Fields] OR “Pediatric index of mortality” OR “PIM”[All Fields]) AND ((“intensive care units”[MeSH Terms] OR (“intensive”[All Fields] AND “care”[All Fields] AND “units”[All Fields]) OR “intensive care units”[All Fields] OR “icu”[All Fields]) AND (“patient s”[All Fields] OR “patients”[MeSH Terms] OR “patients”[All Fields] OR “patient”[All Fields] OR “patients s”[All Fields])) AND (“mortality”[MeSH Terms] OR “mortality”[All Fields] OR “mortalities”[All Fields] OR “mortality”[MeSH Subheading]). A search was restricted to human subjects only. The year of publication filter was 1996 and after.

### PICO Question

#### Participants

We included studies on patients admitted to PICU for any conditions.

#### Prognostic Tests

Studies with PRISM-III/IV, PIM-3, and PELOD-2 model

**Comparator:** Threshold values reported in the published articles
**Outcome:** The outcome assessed was mortality. Mortality was defined as death at hospital or follow-up.

### Eligibility Criteria

#### Inclusion Criteria

Study design: All studies evaluating the accuracy of PELOD-2, PIM-3, or PRISM-III/IV scores to predict outcomes in pediatric patients admitted to the ICU. These prognostic models should aim to predict mortality at any time point in PICU patients aged <18 years.

#### Exclusion Criteria

Not reporting relevant outcome (mortality) in PICU patients, case reports, review articles.

### Data Collection

Two independent authors screened the title, shortlisted the relevant articles, and extracted the data from the potentially eligible articles that meet the inclusion criteria of the study. Disagreements were resolved by discussion. The data extraction form consisted of the following information: first author of the published article, publication year, details of participants, sample size, details of prediction scoring system, settings, and country from where the data were reported.

### Statistical Analysis

STATA software version 13 was used to analyze the data. A random-effects model was used to calculate pooled sensitivity and pooled specificity with a 95% confidence interval (CI), and summary area under the curve with 95% CI. Heterogeneity was calculated with the *I*^2^ statistic. The *I*^2^ = 50% was considered as significant heterogeneity. The methodological quality of studies was assessed using the PROBAST (Prediction Model Risk of Bias Assessment Tool) (12) on four domains: (a). participants selection, (b). prediction selection and measurement, (c). outcome definition and measurement, and (d). statistical analysis which consists of a total of 20 signaling questions to assess the risk of bias. The signaling questions are rated as yes, probably yes, no, probably no, or no information. In case all signaling questions are rated yes or probably yes, then the study is rated as low risk of bias, whereas no or probability no on one or more questions was rated as potential risk of bias. The studies in which there was insufficient information to judge on one or more question were rated as unclear risk of bias. All the studies were rated as low risk of bias for mortality in consideration that there would be no bias in the measurement.

### GRADE Evidence

An adapted GRADE framework for determining the certainty of evidence in predictive accuracy studies was used (13). The GRADE of evidence was judged using risk of bias, indirectness, inconsistency, impression, publication bias, large effect, and possible cofounding effects.

## Results

### Study Characteristics

**Figure 1:**
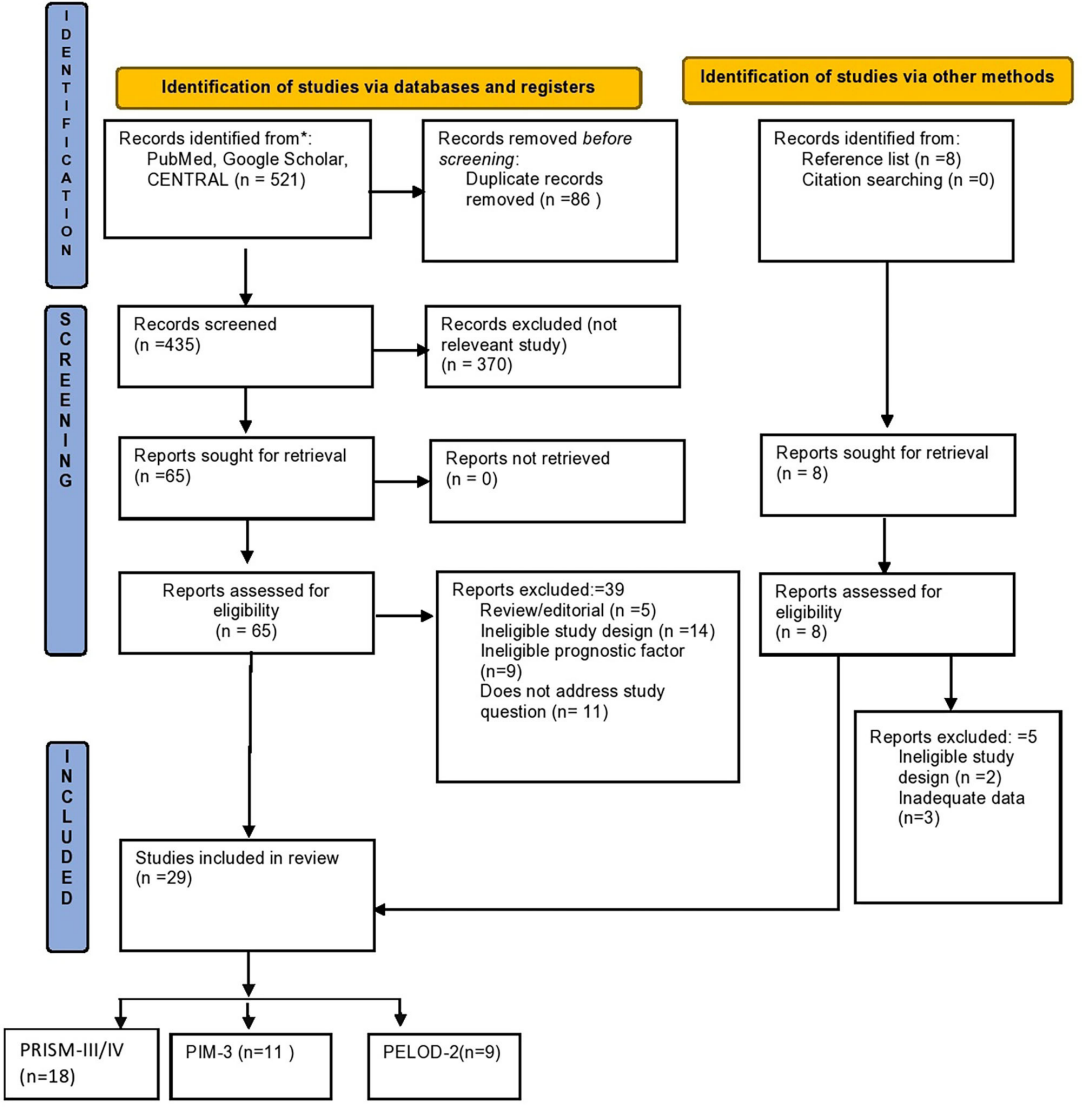
Study flow diagram.

Study characteristics are shown in Table 1. The study flow diagram is shown in Figure 1. A total of 29 studies were included for quantitative synthesis, among them, 18 studies that reported on the scoring systems PRISM-III/IV, (3, 4, 11, 14–21, 23–27, 37), 11 studies that reported data on PIM-3 (3, 4, 7, 14, 16, 18, 20, 28–31), and nine studies that reported data on PELOD-2 (5, 14, 16, 28, 32–36). Four studies were reported from India (4, 7, 17, 34), two from Australia (20, 32), two from China (20, 32), two from Egypt (15, 18), one from Pakistan (19, 26), two from Korea (14, 36), one from Mexico (21), one from Singapore (28), one from UAE (29), one from Indonesia (30), one from Africa (33), one from Saudi Arabia (11), two from Turkey (22, 24), one from Sweden (23), one from Brazil (37), one from Switzerland (5), one from Thailand (27), one from Italy (31), and one multi-centric (25).

**Table 1:** Study characteristics of studies included in the systematic review and meta-analysis.

**Study no**.	**References**	**Country**	**Study design**	**Study period**	**Total sample size**	**Survive**	**Death**	**Mean/** **median age**	**Median length**	**Associated disease**
	**PRISM-III/IV**									
1.	Tyagi et al. (4) (III)	India	Not included	18 months	350	212	138	12 months	5 days	
2.	Jung et al. (14) (III)	Korea	Retrospective	March 2009 and February 2015	503	403	100	NR	NR	
3.	Hamshery et al. (15) (III)	Egypt	Retrospective	January to December 2011	237	143	94	12 months	7 days	
4.	Nienderwanger et al. (16) (III)	Australia	Retrospective	NR	398	342	56	30 months	NR	Cancer
5.	Kaur et al. (17) (III)	India	NR	January 2014 to June 2015	486	335	151	NR	NR	
6.	Abdelkader et al. (18) (III)	Egypt	Cohort study	January to December 2016	68	56	12	7.6 ± 5.3 years	9.8 ± 7	
7.	Mirza et al. (19) (III)	Pakistan	Prospective cohort	December 2017 to June 2019	407	255	152	27 ± 33 months	80.15 ± 15 h	
8.	Choi et al. (20) (III)	China	Cohort	April 2001 to March 2003	303	295	8	2 years	3 days	
9.	León et al. (21) (III)	Mexico	Prospective cohort	Not mentioned	170	128	42	5.3 years	6 days	
10.	Albuali et al. (11) (III)	Saudi Arabia	Retrospective	January 2015 to December 2019	400	335	65	NR	12 days	
11.	Dursun et al. (22) (III)	Turkey	Retrospective	January 1, 2015 and January 1, 2018	48	11	37	77 months	5 days	cancer
12.	Ehinger et al. (23) (III)	Sweden	Retrospective	1 January 2006 through 31 March 2016	2,434	2,308	126	NR	NR	Mitochondrial disorder
13.	Leal et al. (37) (IV)	Brazil	Ambispective cohort	March 1, 2017 to April 30, 2019 (prospective) and March 1, 2017 to November 1, 2014 (retrospective)	1,003	875	128	93 months	NR	Cancer
14.	Dursun et al. (24) (III)	Turkey	Retrospective cohort	August 2004 and August 2007	36	16	20	5 years	4 days.	
15.	Jacobs et al. (25) (III)	Multicentric	Prospective study	November 2013–December 2016	1,428	1,360	68	1 year	2 days	
16.	Bilan et al. (26)	Pakistan	Prospective	March 2006 to April 2007	221	20	201	31.64 months	5.11 days	
17.	Ruangnapa et al. (27) (III)	Thailand	Retrospective	November 2013 to December 2016	588	82	506		3.5 day	
18.	Ramazani et al. (3) (III)	Iran	Prospective	July 2014 to October 2015	90	74	16	7.80 ± 4.43 years	3.65 ± 3.95 days	
	**PIM-3**									
19.	Niederwanger et al. (16)	Australia	Retrospective	NR	398	344	54	30 Months	NR	
20.	Jung et al. (14)	Korea	Retrospective	March 2009 and February 2015	503	403	100	NR	NR	
21.	Tyagi et al. (4)	India	Not included	18 months	350	212	138	12 months	5 days	
22.	Wong et al. (28)	Singapore	Prospective cohort	1 April 2015 to 31 March 2016	570	535	35	NR	28 days	
23.	Malhotra et al. (29)	UAE	Retrospective cohort	January 2016 to October 2018	583	537	46	37 months	NR	
24.	Sari et al. (30)	Indonesia	Prospective cohort	Feb to April 2016	69	41	28	89 months	NR	cancer
25.	Abdelkader et al. (18)	Egypt	Cohort study	January to December 2016	68	56	12	7.6 ± 5.3 years	9.8 ± 7 days	
26.	Sankar et al. (7)	India	Cohort	September 2015 to July 2016	202	133	69	3 years	NR	
27.	Jacobs et al. (25) (III)	Multicentric	Retrospective	November 2013–December 2016	1,428	1360	68	1 year	2 days	
28.	Wolfler et al. (31)	Italy	Retrospective	January 2010 to October 2014	11,109	10677	432	46.3 Months	2 days	
29.	Ramazani et al. (3)	Iran	Prospective	July 2014 to October 2015	90	74	16	7.80 ± 4.43 years	3.65 ± 3.95 days	
	**PELOD-2**									
1	Zhong et al. (32)	China	Retrospective	June 1, 2016 to June 1, 2018	516	488	28	8 months	24 h	
2	Nawawy et al. (33)	Africa	Prospective cohort study	July 2015 and April 2016	190	140	50	6 months	days	
3	Deshmukh et al. (34)	India	NR	NR	129	109	20	67 months	NR	
4	Schlapbach et al. (35)	Australia	Cohort study	NR	2,594	280	94	13 years	>3 days	
5	Nienderwanger et al. (16)	Australia	Retrospective	NR	398	342	56	30 Months	NR	Cancer
6	Wong et al. (28)	Singapore	Prospective cohort	1 April 2015 to 31 March 2016	570	535	35	NR	28 days	
7	Kim et al. (36)	Korea	Retrospective	November 2012 to May 2018	960	876	84	15.5 months	NR	
8	Karam et al. (5)	Switzerland	Prospective	NR	443	324	119	1 year	10 days	
9	Ramazani et al. (3)	Iran	Prospective	July 2014 to October 2015	90	74	16	7.80 ± 4.43 years	3.65 ± 3.95 days	

**Figure 2:**
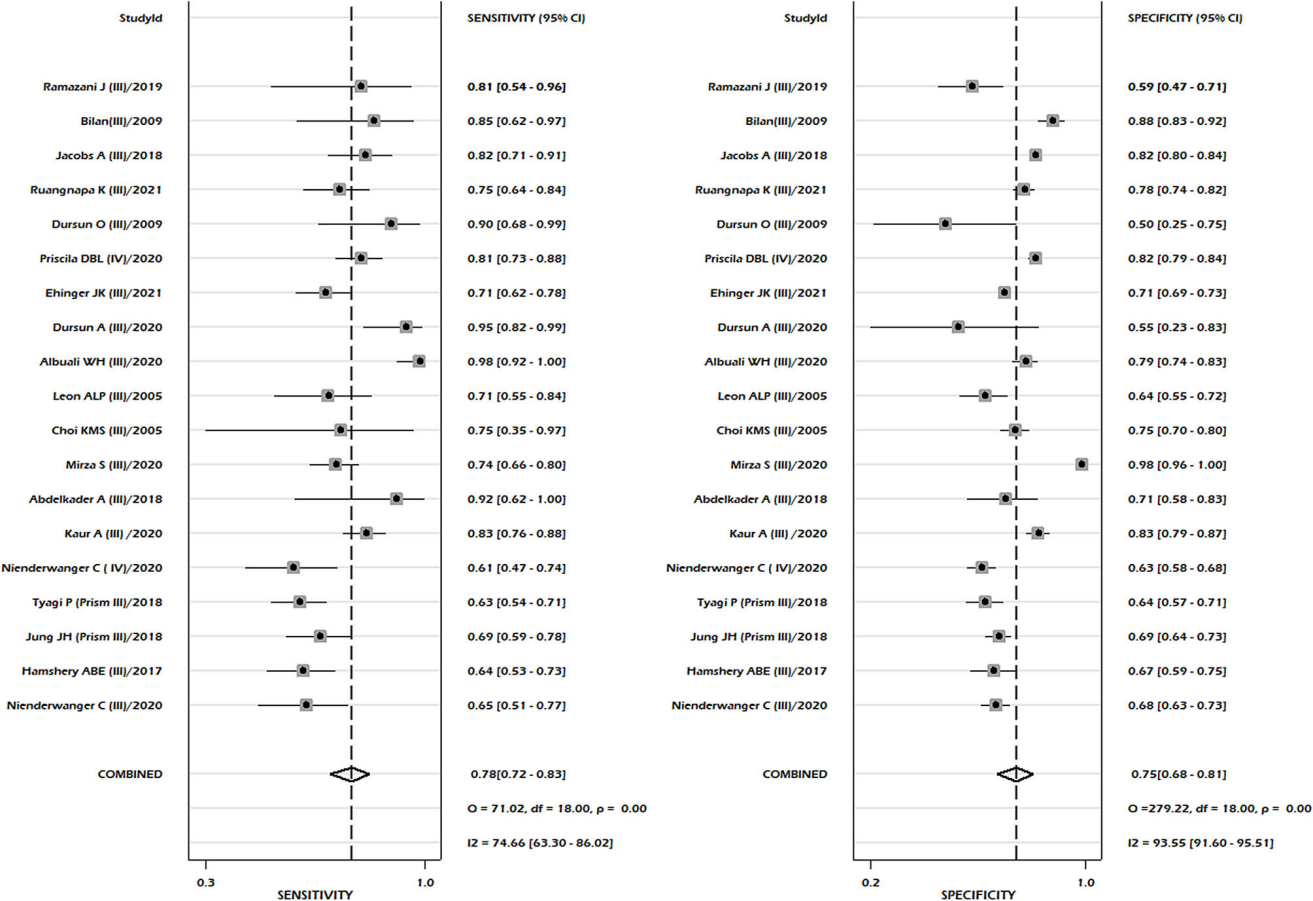
Pooled sensitivity and pooled specificity for PRISM-III/IV.

A total of 18 studies reported sufficient data to compute pooled sensitivity and pooled specificity for the PRISM-III/IV scoring system. Sixteen studies were conducted in PRISM-III and two studies used PRISM-IV models. The meta-analysis of combined PRISM-III/IV studies showed pooled sensitivity of 0.78, 95% CI: 0.72–0.83, and a pooled specificity of 0.75, 95% CI: 0.68–0.81 (Figure 2). Our pooled analysis observed good ability of test performance of PRISM-III/IV (diagnostic odds ratio 11, 95% CI; 7–18).

**Figure 3:**
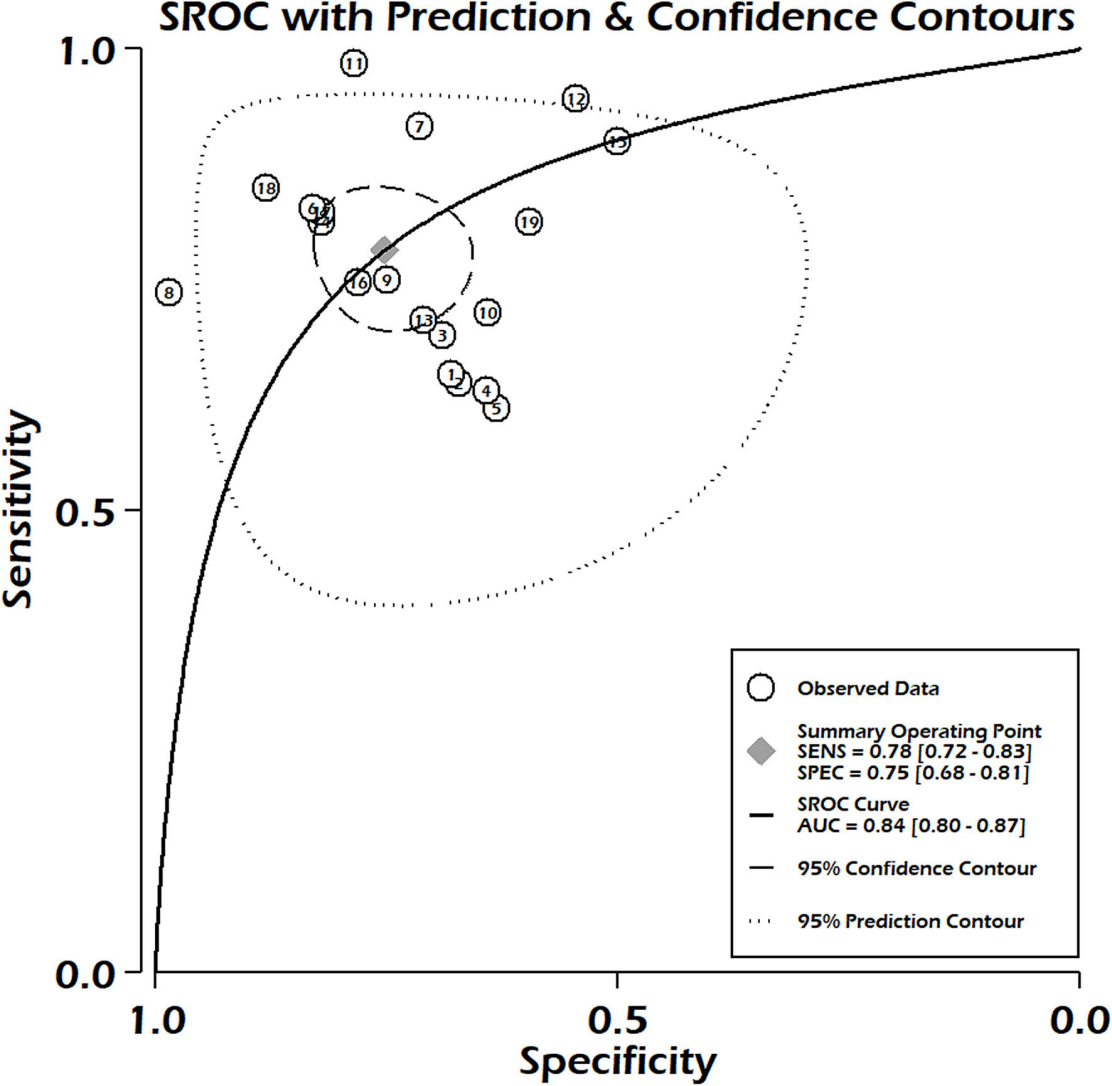
Summary receiver operating characteristic curve for PRISM-III/IV.

Studies including only PRISM-III reported pooled sensitivity of 0.79, 95% CI 0.72–0.85, and specificity 0.75, 95% CI 0.68–0.82. The summary area under the curve suggested 84% discriminatory power of PRISM-III/IV for mortality (SROC 0.84, 95% CI: 0.80–0.87) (Figure 3). We could not compute the pooled sensitivity and pooled specificity of the PRISM-IV due to the small number of studies, insufficient for subgroup analysis. There was significant heterogeneity between the studies for pooled sensitivity (*p* < 0.001) and specificity (*p* < 0.001) analyses (Figure 2), with no significant publication bias (*p* = 0.81) (Supplementary Figure 1). We observed moderate to high risk of bias in the risk of bias analysis between studies, which was mainly in the statistical analysis domain (Supplementary Figures 2A,B). Our meta-regression analysis did not observe the significant influence of differences in mortality rates among different populations, study design, mean age of PICU patients, female gender, and setting (specialized children hospital/tertiary care hospitals), study period, and length of hospital stay on the discriminatory and predictive performance of PRISM III/IV (Supplementary Figure 3). The level of evidence using GRADE criteria observed very low certainty of evidence (Supplementary Table 1).

**Figure 4:**
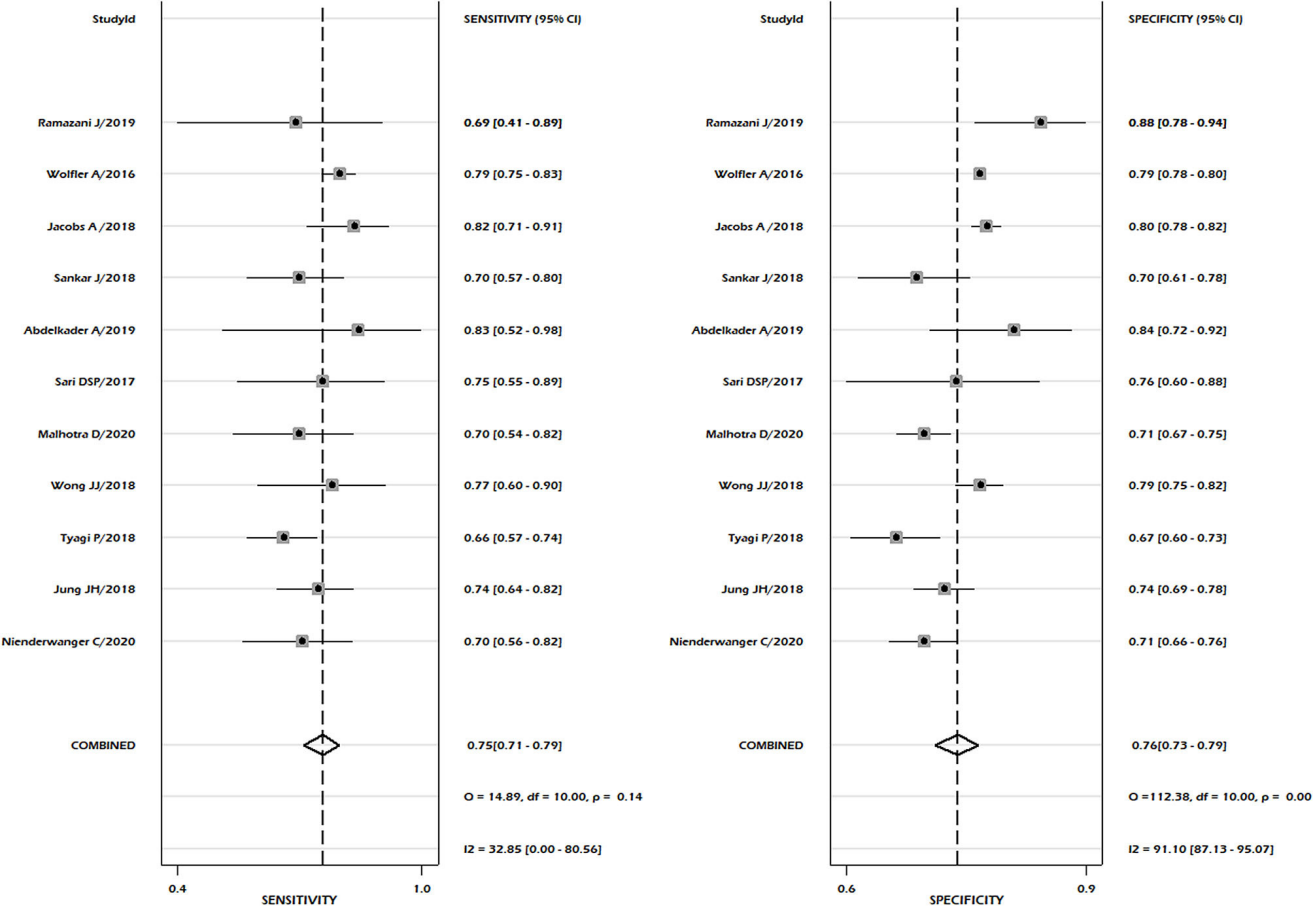
Pooled sensitivity and pooled specificity for PIM-3.

**Figure 5:**
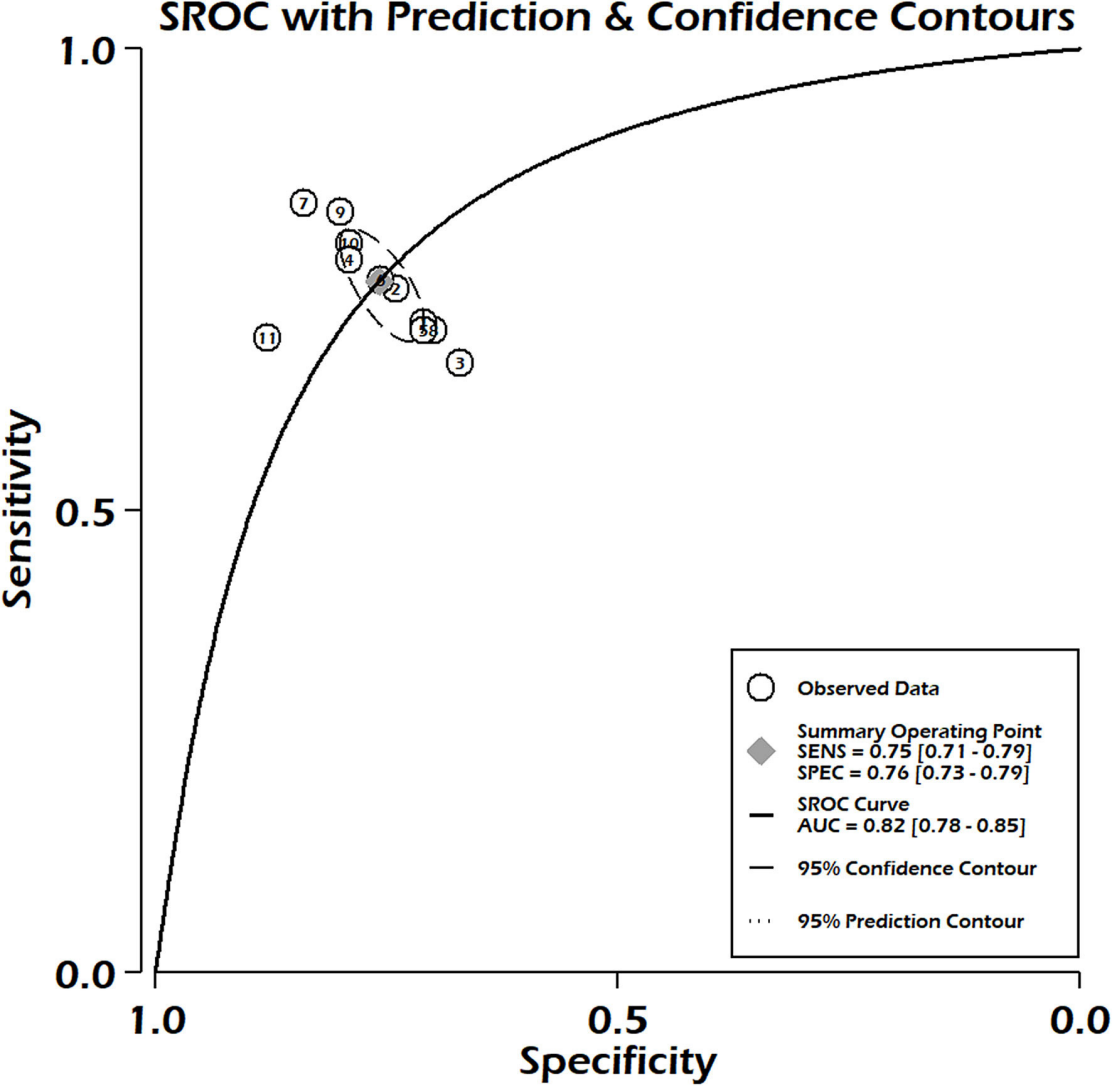
Summary receiver operating characteristic curve for PIM-3.

In the case of PIM-3, 11 studies fulfilled the inclusion criteria to determine pooled sensitivity and pooled specificity. We reported pooled sensitivity of 0.75 (95% CI: 0.71–0.79) and combined specificity of 0.76 (95% CI: 0.73–0.79) (Figure 4). No significant heterogeneity was observed for both sensitivity (*p* = 0.14, *I*^2^ = 32.85), but significant heterogeneity was noted in pooled specificity (*p* < 0.001, *I*^2^ = 91%) (Figure 4). Publication bias was absent in the combined sensitivity and specificity (*p* = 0.36) (Supplementary Figure 4). The summary area under the curve indicated that the PIM-3 scoring system had 82% prediction power to predict mortality (SROC 0.82, 95% CI: 0.78–0.85) (Figure 5). Our pooled analysis observed good ability of test performance for PIM-3 (diagnostic odds ratio 9, 95% CI; 7–13). In the assessment of the methodological quality of studies using the PROBAST tool, we observed moderate to high risk of bias mainly due to inadequate statistical analysis (Supplementary Figures 5A,B). The meta-regression analysis did not observe the significant effect of differences in mortality rates and length of stay on pooled effect size (Supplementary Figure 6). The certainty of evidence was moderate for sensitivity and very low for specificity (Supplementary Table 2).

**Figure 6:**
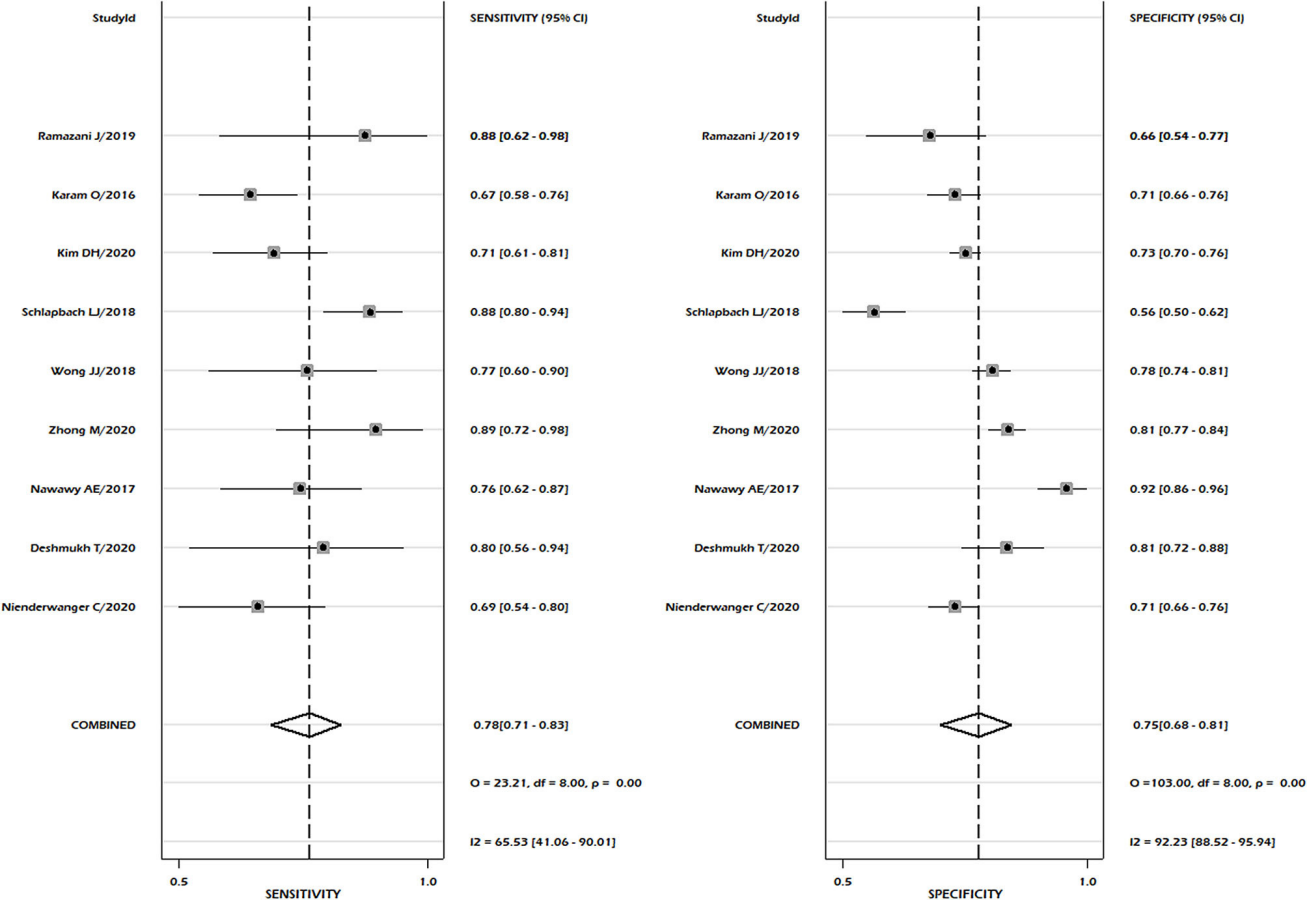
Pooled sensitivity and pooled specificity for PELOD-2.

**Figure 7:**
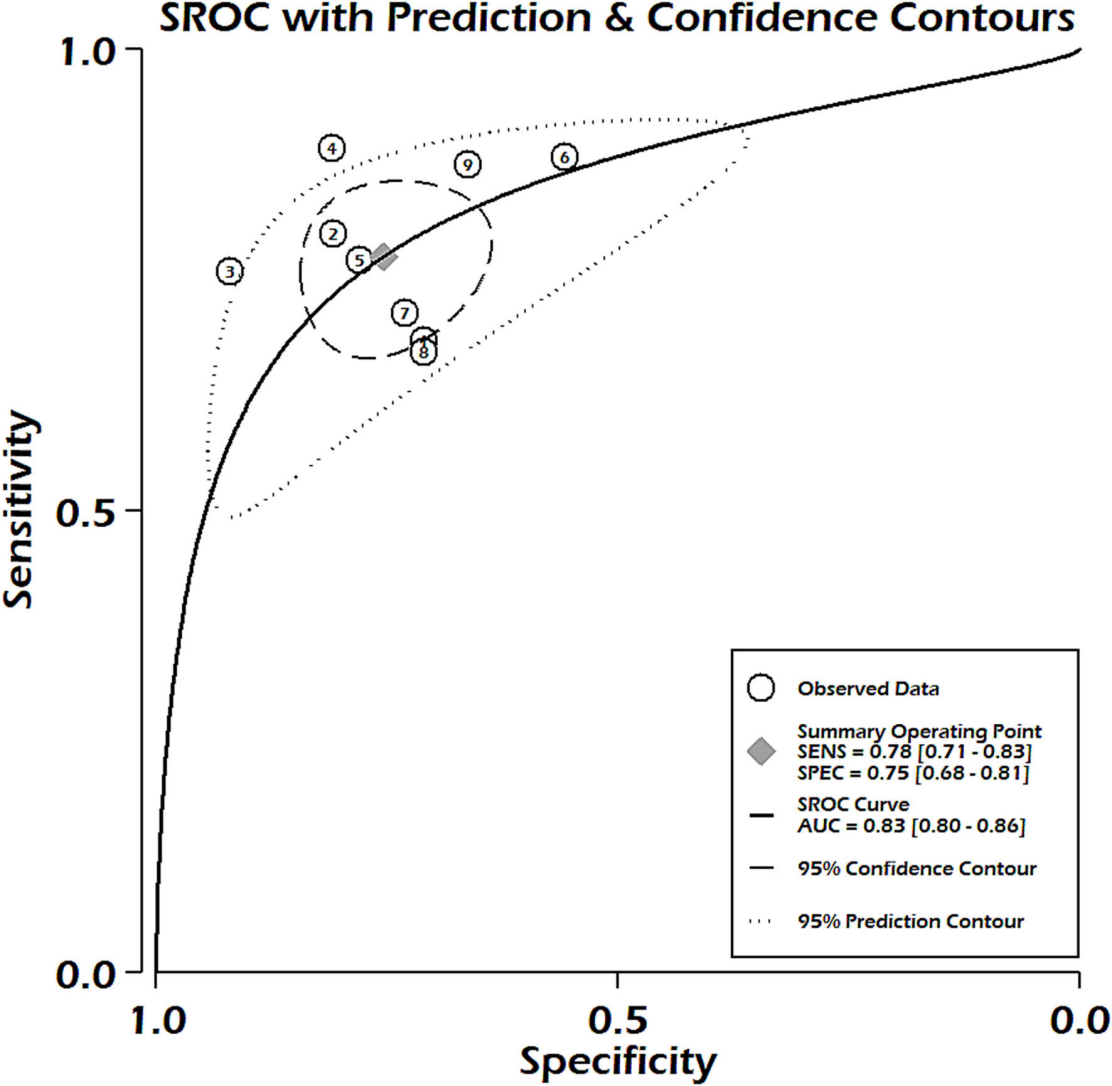
Summary receiver operating characteristic curve for PELOD-2.

Nine studies reported sufficient data for pooled analysis of the sensitivity and specificity of the PELOD-2 scoring system. Pooled analysis showed a pooled sensitivity of 0.78, 95% CI 0.71–0.83, and pooled specificity of 0.75, 95% CI 0.68–0.81 (Figure 6). Heterogeneity was significant for both sensitivity and specificity (*p* < 0.001, *I*^2^ = 65.53% for sensitivity and 92.3% for specificity). Discriminatory performance was observed good as depicted by SROC 0.83; 95% CI 0.80–0.86 (Figure 7), with no statistically significant publication bias (*p* = 0.07) (Supplementary Figure 7). Our pooled analysis observed good ability of test performance for PIM-3 (diagnostic odds ratio 11, 95% CI; 7–17). Methodological quality was moderate to high (Supplementary Figures 8A,B). Our meta-regression analysis did not observe the significant influence of differences in mortality rates, study design, mean age of PICU patients, female gender, study period, and length of hospital stay on the discriminatory and predictive performance of PELOD-2 (Supplementary Figure 9).

## Discussion

In this study, we investigated the predictive accuracy and discriminating power of commonly used scoring systems such as PRISM-III/IV, PELOD-2, and PIM-3 to predict mortality risk in patients admitted to PICU. In China, mortality rates associated with PICU admission are approximately two or three times higher than in America and Europe. It is a need of the hour to identify predictor or prediction models of death in the PICU. There are constant explorations of death risk prediction score for providing optimal management to PICU patients with available resources.

Accurate and reliable information about predicted mortality improves communication with patients about possible prognoses and optimal stratification of patients at risk. These three scoring systems have potential to provide the predictive accuracy for prognosis in PICU patients.

We observed the evidence for good performance of these models; however, risk of bias assessment showed that evidence is with moderate to high risk of bias among studies. This bias was observed mainly due to inadequate presentation and reporting of statistical analysis, and failure to conduct the internal and external validation of models. The calibration of models is an essential component for evaluation of a test model; however, in our analysis, a total of 36% for PRISM-III/IV, 33% for PELOD-2, and 9% for PIM-3 models did not report the calibration of the model, which leads to bias in the statistical analysis domain. In the case of event per variable, 68% of studies in PRISM-III/IV, 88% of studies in PELOD-2, and 72% of studies in PIM-3 had <100 death events, resulting in high risk of bias as per PROBAST tool, which resulted into a risk of over fitting of the model in the validation studies. The most commonly used method to report calibration was the Hosmer–Lemeshow test, whereas this test is limited by neither the presence nor the magnitude of miscalibration (12). To overcome this, it is recommended to present the calibration plot, but most of the studies considered in the present meta-analysis did not present the same.

The development of valid and reliable models for predicting mortality in PICU patients is an ongoing practice. We noted that the PRISM-III/IV score had the best predictive accuracy and discrimination in an individual patient (sROC 0.84), closely followed by PELOD-2 and PIM-3. We found the almost similar discriminatory performances of these scoring systems.

Each of the prediction scores is applied at a specific timeframe in which reliable and optimal performance of prediction is to be expected. In the case of PRISM-III/IV scores, the optimal time point for prediction is after 24 h, while PIM-3 scores show the best performance and discrimination during the early hour after admission. A delayed timeframe that occurs in the case of PRISM-III/IV carries a risk of a patient dying before the assessment of PRISM-III/IV score, which could provide the probability of prognosis (38). On the other hand, the examination in the first few hours may result in an inaccurate predictive ability of prognosis. A study, assessing the predictive ability of PRISM-III, PIM-3, and PELOD-2 in a PICU setting, demonstrated that PIM-3 had better discrimination power and calibration compared to PRISM-III and PELOD-2 (3).

The PELOD-2 score may serve as an optimal measure to monitor the development of disease conditions and predict the outcome when evaluated in continuous time intervals at the time of disease progression (39). A study reported by Zhong et al. (32) reported that the PELOD-2 score was effective to assess the prognosis of PICU patients with sepsis and has shown an excellent discriminatory power with 0.916. On the other hand, PRISM-III/IV score and PELOD-2 performance becomes better when sepsis is pronounced (16). Another study reported by Mathews et al. showed that the PELOD-2 score of over 20 was able to predict mortality in 72.2% of PICU patients, and the cut-off score >16 showed a sensitivity of 100% and specificity of 54.1% (40). The study by Karam et al. (5) further showed the good calibration of the model, with a day 1 PELOD-2 AUC of 0.76 (95 CI 0.71–0.81) and Hosmer-Lemeshow test *p* = 0.76. Good Calibration and discrimination of PELOD-2 were also reported in the study by Deshmukh et al. (34) (AUC = 0.93) and chi-square test for goodness of fit p = 0.45 in PICU patients with sepsis, further confirming the validity and reliability of the model.

A study on large sample size (21,335 subjects in the entire cohort) published by Christoper et al. (41) conducted a retrospective, single-center cohort derived from structured electronic health record data in the large quaternary PICU at a freestanding, university-affiliated children's hospital. The findings of this study demonstrated good to excellent discrimination measured by area under the curve (electronic-PRISM-IV had an area under the curve of 0.90 (95% CI 0.86–0.94), and PELOD-2 0.97 (95% CI 0.96–0.98) of PELOD-2, further strengthening the validity and reliability of scoring systems for accurate prediction of mortality in PICU patients. However, the findings of this study were largely limited by inclusion of only structured electronic data. This study also reported that bias associated with entry of diagnostic codes by physician could not be excluded.

Our meta-regression analysis was to explore the source of variation on the discriminatory and predictive performance indicating the need of well-designed studies with additional clinically relevant variables to explore the source of heterogeneity between the studies.

Regarding the certainty of evidence using GRADE analysis, we rated our certainty of evidence at very low for PRISM-III/IV, low for PELOD-2, and moderate for PIM-3 for predicting mortality in PICU patients. This means that the true effects are likely to be close to the estimated prognostic significance, but there are possibilities that it is substantially different.

## Limitation

This study has several limitations. A high degree of heterogeneity was noted in the pooled analysis, which can originate from differences between study population, setting, and methodological quality of the studies. Considering the heterogeneity across the studies, further research will be necessary to obtain homogenous findings. A large sample size study reported by Christoper et al. could not be included in the analysis due to insufficient required data that resulted in the underestimation or overestimation of some of the studied scoring systems. Studies included in the meta-analysis were conducted in a wide range of conditions and settings leading to heterogeneity in the study findings.

## Conclusion

PIM-3, PELOD-2, and PRISM III/IV demonstrated good discriminatory power for mortality prediction in PICU patients with low to moderate quality of evidence. Further better-designed studies are needed to provide a better and accurate judgment of the performances of these models.

## Data Availability Statement

The raw data supporting the conclusions of this article will be made available by the authors, without undue reservation.

## Author Contributions

YS and JJ conceived and designed the study, involved in literature search, data collection, and reviewed and edited the manuscript. YS analyzed the data. JJ wrote the paper. All authors read and approved the final manuscript.

## Conflict of Interest

The authors declare that the research was conducted in the absence of any commercial or financial relationships that could be construed as a potential conflict of interest.

## Publisher's Note

All claims expressed in this article are solely those of the authors and do not necessarily represent those of their affiliated organizations, or those of the publisher, the editors and the reviewers. Any product that may be evaluated in this article, or claim that may be made by its manufacturer, is not guaranteed or endorsed by the publisher.

## References

[1] Toltzis P Soto-Campos G Shelton CR Kuhn EM Hahn R Kanter RK. Evidence-based pediatric outcome predictors to guide the allocation of critical care resources in a mass casualty event. Pediatr Crit Care Med. (2015) 16:e207–16. 10.1097/PCC.0000000000000481 26121100

[2] Li J-J Chen Y-F Lin Y-X. [Investigation of disease spectrum in the PICU of Shengjing Hospital of China Medical University between 2005 and 2012]. Zhongguo Dang Dai Er Ke Za Zhi Chin J Contemp Pediatr. (2013) 15:472–76. 23791065

[3] Ramazani J Hosseini M. Comparison of the predictive ability of the pediatric risk of mortality iii, pediatric index of mortality3, and pediatric logistic organ dysfunction-2 in medical and surgical intensive care units. J Compr Pediatr. (2019) 10:e82830. 10.5812/compreped.82830

[4] Tyagi P Tullu MS Agrawal M. Comparison of pediatric risk of mortality iii, pediatric index of mortality 2, and pediatric index of mortality 3 in predicting mortality in a pediatric intensive care unit. J Pediatr Intensive Care. (2018) 7:201–6. 10.1055/s-0038-1673671 31073495 PMC6506681

[5] Karam O Demaret P Duhamel A Shefler A Spinella PC Stanworth SJ. Performance of the PEdiatric Logistic Organ Dysfunction-2 score in critically ill children requiring plasma transfusions. Ann Intensive Care. (2016) 6:98. 10.1186/s13613-016-0197-6 27714707 PMC5053948

[6] Shann F Pearson G Slater A Wilkinson K. Paediatric index of mortality (PIM): a mortality prediction model for children in intensive care. Intensive Care Med. (1997) 23:201–7. 10.1007/s001340050317 9069007

[7] Sankar J Gulla KM Kumar UV Lodha R Kabra SK. Comparison of outcomes using pediatric index of mortality (PIM)−3 and PIM-2 models in a pediatric intensive care unit. Indian Pediatr. (2018) 55:972–4. 10.1007/s13312-018-1421-2 30587646

[8] Straney L Clements A Parslow RC Pearson G Shann F Alexander J Slater A ANZICS Paediatric study group and the paediatric intensive care audit network. paediatric index of mortality 3: an updated model for predicting mortality in pediatric intensive care^*^. Pediatr Crit Care Med. (2013) 14:673–81. 10.1097/PCC.0b013e31829760cf 23863821

[9] Pollack MM Patel KM Ruttimann UE. PRISM III: an updated pediatric risk of mortality score. Crit Care Med. (1996) 24:743–52. 10.1097/00003246-199605000-00004 8706448

[10] Leteurtre S Duhamel A Salleron J Grandbastien B Lacroix J Leclerc F Groupe Francophone de Réanimation et d'Urgences Pédiatriques (GFRUP). PELOD-2: an update of the PEdiatric logistic organ dysfunction score. Crit Care Med. (2013) 41:1761–73. 10.1097/CCM.0b013e31828a2bbd 23685639

[11] Albuali WH Algamdi AA Hasan EA Al-Qahtani MH Yousef AA Al Ghamdi MA. Use of a mortality prediction model in children on mechanical ventilation: a 5-year experience in a tertiary university hospital. J Multidiscip Healthc. (2020) 13:1507–16. 10.2147/JMDH.S282108 33204099 PMC7667207

[12] Moons KGM Wolff RF Riley RD Whiting PF Westwood M Collins GS. PROBAST: a tool to assess risk of bias and applicability of prediction model studies: explanation and elaboration. Ann Intern Med. (2019) 170:W1–33. 10.7326/M18-1377 30596876

[13] Foroutan F Guyatt G Zuk V Vandvik PO Alba AC Mustafa R. GRADE guidelines 28: use of GRADE for the assessment of evidence about prognostic factors: rating certainty in identification of groups of patients with different absolute risks. J Clin Epidemiol. (2020) 121:62–70. 10.1016/j.jclinepi.2019.12.023 31982539

[14] Jung JH Sol IS Kim MJ Kim YH Kim KW Sohn MH. Validation of pediatric index of mortality 3 for predicting mortality among patients admitted to a pediatric intensive care unit. Acute Crit Care. (2018) 33:170–7. 10.4266/acc.2018.00150 31723881 PMC6786694

[15] Hamshary AAEE Sherbini SAE Elgebaly HF Amin SA. Prevalence of multiple organ dysfunction in the pediatric intensive care unit: pediatric Risk of Mortality III versus pediatric logistic organ dysfunction scores for mortality prediction. Rev Bras Ter Intensiva. (2017) 29:206–12. 10.5935/0103-507X.20170029 28977260 PMC5496755

[16] Niederwanger C Varga T Hell T Stuerzel D Prem J Gassner M. Comparison of pediatric scoring systems for mortality in septic patients and the impact of missing information on their predictive power: a retrospective analysis. PeerJ. (2020) 8:e9993. 10.7717/peerj.9993 33083117 PMC7543722

[17] Kaur A Kaur G Dhir SK Rai S Sethi A Brar A. Pediatric risk of mortality III Score—predictor of mortality and hospital stay in pediatric intensive care unit. J Emerg Trauma Shock. (2020) 13:146–50. 10.4103/JETS.JETS_89_19 33013095 PMC7472814

[18] Abdelkader A Shaaban M Zahran M. Using two scores for the prediction of mortality in pediatric intensive care units. Al-Azhar Assiut Med J. (2018) 16:349. 10.4103/AZMJ.AZMJ_48_1833996681

[19] Mirza S Malik L Ahmed J Malik F Sadiq H Ali S. Accuracy of pediatric risk of mortality (PRISM) III score in predicting mortality outcomes in a pediatric intensive care unit in karachi. Cureus. (2020) 12:e7489. 10.7759/cureus.7489 32368422 PMC7193246

[20] Choi KMS Ng DKK Wong SF Kwok KL Chow PY Chan CH. Assessment of the pediatric index of mortality (PIM) and the pediatric risk of mortality (PRISM) III score for prediction of mortality in a paediatric intensive care unit in Hong Kong. Hong Kong Med J Xianggang Yi Xue Za Zhi. (2005) 11:97–103. 15815062

[21] De León ALP-P Romero-Gutiérrez G Valenzuela CA González-Bravo FE. Simplified PRISM III score and outcome in the pediatric intensive care unit. Pediatr Int Off J Jpn Pediatr Soc. (2005) 47:80–3. 10.1111/j.1442-200x.2004.01997.x 15693872

[22] Dursun A Özsoylu S Akyildiz BN. Outcomes and prognostic factors for pediatric cancer patients admitted to an intensive care unit in a university hospital. Turk J Pediatr. (2020) 62:252–8. 10.24953/turkjped.2020.02.011 32419417

[23] Ehinger JK Karlsson M Sjövall F Leffler M McCormack SE Kubis SE. Predictors of outcome in children with disorders of mitochondrial metabolism in the pediatric intensive care unit. Pediatr Res. (2021)1–7. 10.1038/s41390-021-01410-z 33627817 PMC7903037

[24] Dursun O Hazar V Karasu GT Uygun V Tosun O Yesilipek A. Prognostic factors in pediatric cancer patients admitted to the pediatric intensive care unit. J Pediatr Hematol Oncol. (2009) 31:481–4. 10.1097/MPH.0b013e3181a330ef 19564740

[25] Jacobs A Flechet M Vanhorebeek I Verstraete S Ingels C Casaer MP. Performance of pediatric mortality prediction scores for PICU mortality and 90-day mortality. Pediatr Crit Care Med. (2019) 20:113–9. 10.1097/PCC.0000000000001764 30362989

[26] Bilan N Galehgolab BA Emadaddin A Shiva S. Risk of mortality in pediatric intensive care unit, assessed by PRISM-III. Pak J Biol Sci PJBS. (2009) 12:480–5. 10.3923/pjbs.2009.480.485 19579995

[27] Ruangnapa K Sucheewakul S Liabsuetrakul T McNeil E Lim K Anantaseree W. Validation of a modified pediatric risk of mortality III model in a pediatric intensive care unit in Thailand. Pediatr Respirol Crit Care Med. (2018) 2:65. 10.4103/prcm.prcm_11_18

[28] Wong JJ Hornik CP Mok YH Loh TF Lee JH. Performance of the paediatric index of mortality 3 and paediatric logistic organ dysfunction 2 scores in critically ill children. Ann Acad Med Singapore. (2018) 47:285–90. 30242298

[29] Malhotra D Nour N El Halik M Zidan M. Performance and analysis of pediatric index of mortality 3 score in a pediatric ICU in Latifa Hospital, Dubai, UAE. Dubai Med J. (2020) 3:19–25. 10.1159/000505205

[30] Sari DSP Saputra I Triratna S Saleh MI. The pediatric index of mortality 3 score to predict mortality in a pediatric intensive care unit in Palembang, South Sumatera, Indonesia. Paediatr Indones. (2017) 57:164–70. 10.14238/pi57.3.2017.164-70

[31] Wolfler A Osello R Gualino J Calderini E Vigna G Santuz P. The importance of mortality risk assessment: validation of the pediatric index of mortality 3 score. Pediatr Crit Care Med. (2016) 17:251–6. 10.1097/PCC.0000000000000657 26825046

[32] Zhong M Huang Y Li T Xiong L Lin T Li M. Day-1 PELOD-2 and day-1 “quick” PELOD-2 scores in children with sepsis in the PICU. J Pediatr. (2020) 96:660–5. 10.1016/j.jped.2019.07.007 31580846 PMC9432166

[33] El-Nawawy A Mohsen AA Abdel-Malik M Taman SO. Performance of the pediatric logistic organ dysfunction (PELOD) and (PELOD-2) scores in a pediatric intensive care unit of a developing country. Eur J Pediatr. (2017) 176:849–55. 10.1007/s00431-017-2916-x 28492972

[34] Deshmukh T Varma A Damke S Meshram R. Predictive efficacy of pediatric logistic organ dysfunction-2 score in pediatric intensive care unit of rural hospital. Indian J Crit Care Med. (2020) 24:701–4. 10.5005/jp-journals-10071-23528 33024378 PMC7519618

[35] Schlapbach LJ Straney L Bellomo R MacLaren G Pilcher D. Prognostic accuracy of age-adapted SOFA, SIRS, PELOD-2, and qSOFA for in-hospital mortality among children with suspected infection admitted to the intensive care unit. Intensive Care Med. (2018) 44:179–88. 10.1007/s00134-017-5021-8 29256116 PMC5816088

[36] Kim DH Ha EJ Park SJ Jhang WK. Evaluation of the usefulness of red blood cell distribution width in critically ill pediatric patients. Medicine. (2020) 99:e22075. 10.1097/MD.0000000000022075 32899077 PMC7478568

[37] Leal P de B de Araujo OR Petrilli AS da Silva DCB. PRISM 4-C: an adapted PRISM IV algorithm for children with cancer. J Pediatr Hematol Oncol. (2020) 42:e563–8. 10.1097/MPH.0000000000001716 32986390

[38] de Araujo Costa G Delgado AF Ferraro A Okay TS. Application of the Pediatric Risk of Mortality Score (PRISM) score and determination of mortality risk factors in a tertiary pediatric intensive care unit. Clinics. (2010) 65:1087–92. 10.1590/S1807-59322010001100005 21243277 PMC2999700

[39] Leteurtre S Duhamel A Deken V Lacroix J Leclerc F. Daily estimation of the severity of organ dysfunctions in critically ill children by using the PELOD-2 score. Crit Care Lond Engl. (2015) 19:324. 10.1186/s13054-015-1054-y 26369662 PMC4570178

[40] Mathews S Rajan A Soans ST. Prognostic value of rise in neutrophil to lymphocyte ratio (NLR) and platelet to lymphocyte ratio (PLR) in predicting the mortality in paediatric intensive care. Int J Contemp Pediatr. (2019) 6:1052–8. 10.18203/2349-3291.ijcp20191044

[41] Horvat CM Ogoe H Kantawala S Au AK Fink EL Yablonsky E. Development and performance of electronic pediatric risk of mortality and pediatric logistic organ dysfunction-2 automated acuity scores. Pediatr Crit Care Med. (2019) 20:e372–9. 10.1097/PCC.0000000000001998 31397827 PMC7115250

