# Supplementary material for: Meta-Analysis for the Prediction of Mortality Rates in a Pediatric Intensive Care Unit Using Different Scores: PRISM-III/IV, PIM-3, and PELOD-2
Source: Front Pediatr. 2021 Aug 24;9:712276. doi: 10.3389/fped.2021.712276 (PMC8421854; doi:10.3389/fped.2021.712276)
Supplement: Supplementary file 1 [file Data_Sheet_1.docx]

Supplementary figure

Supplementary figure 1: Publication bias for PRISM-III/IV


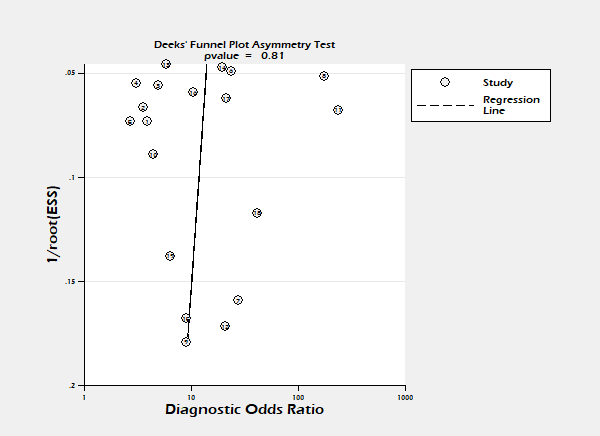


Supplementary Figure 2A


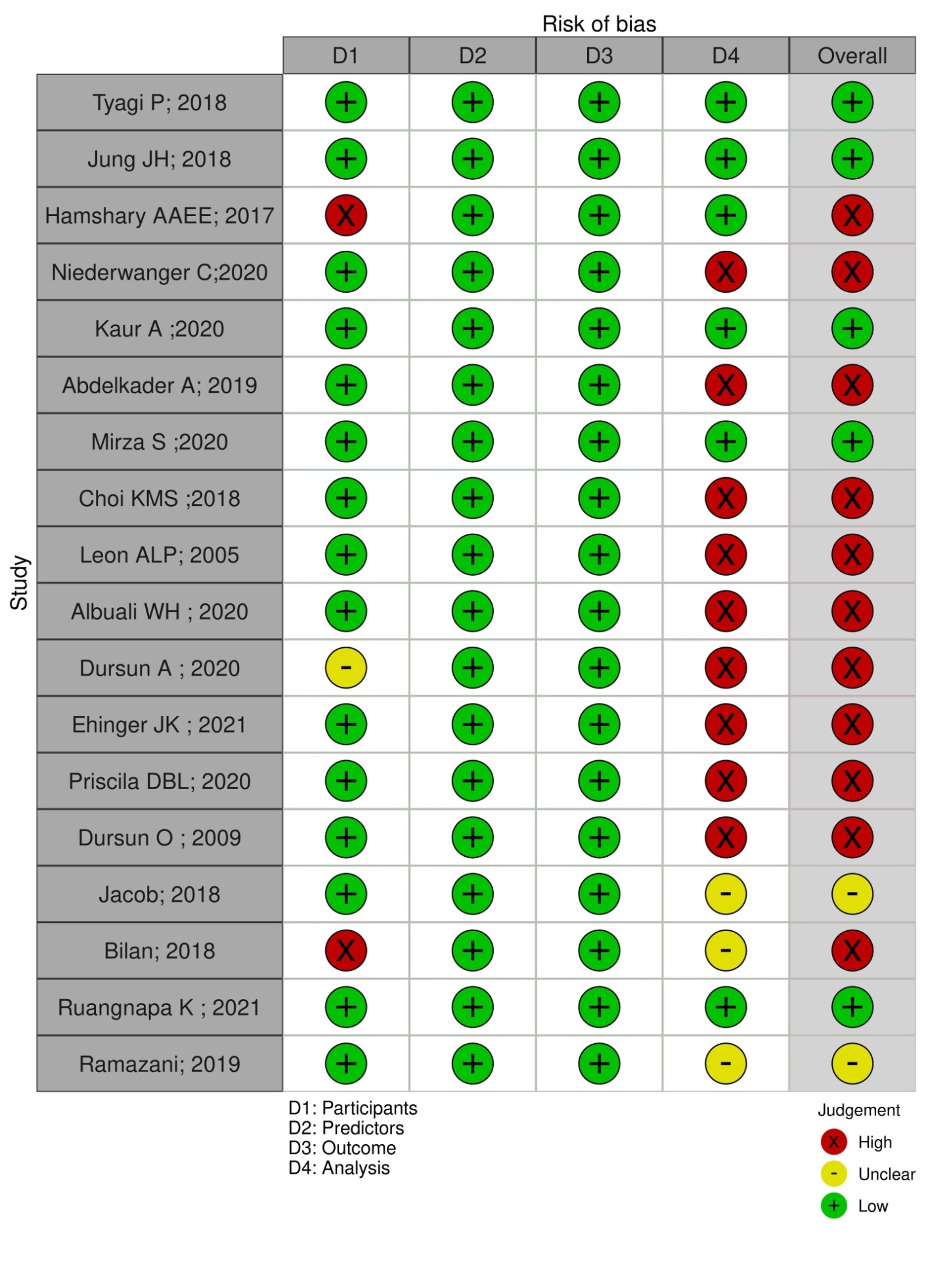


Supplementary Figure 2B


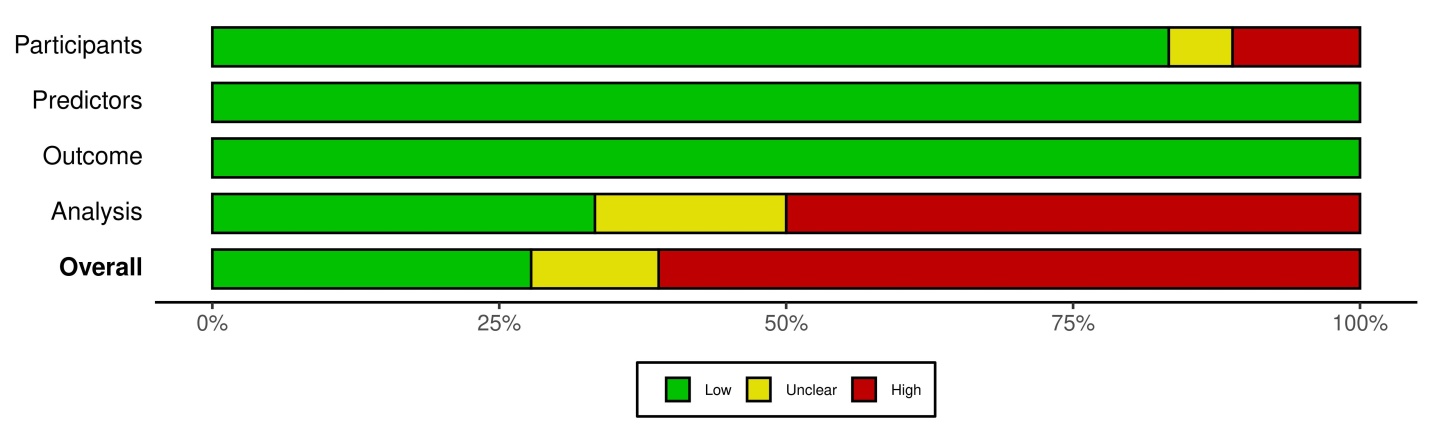


Supplementary Figure 3: Meta-regression analysis to explore the effects of individual score on pooled effect size of PRISM-III/IV scoring system.


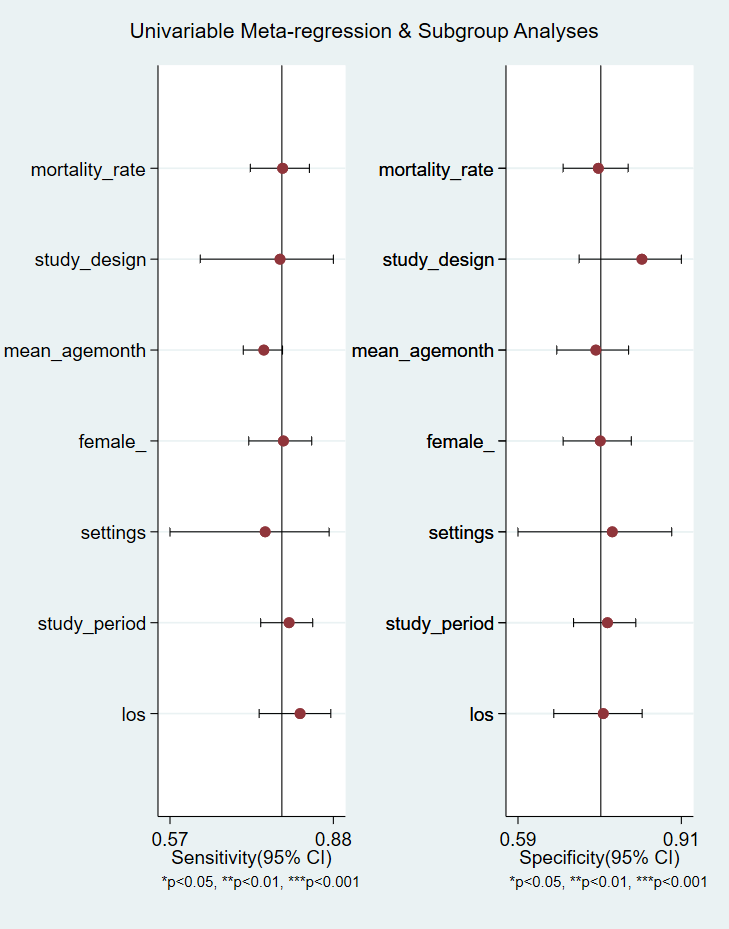


Supplementary Figure 4 : Funnel plot showing publication bias for PIM-3 scoring system


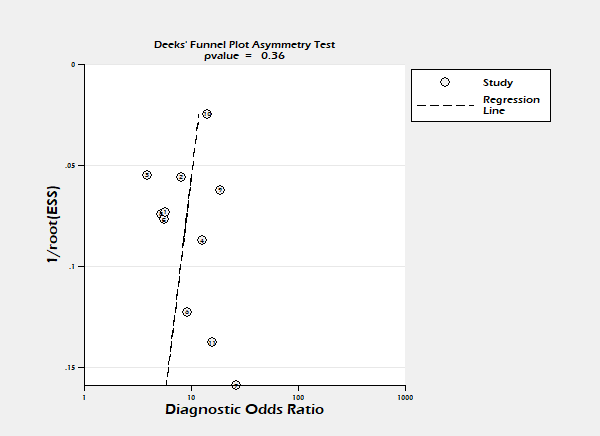


Supplementary Figure 5A : Risk of Bias assessment using PROBAST tool showing bias of individual studies


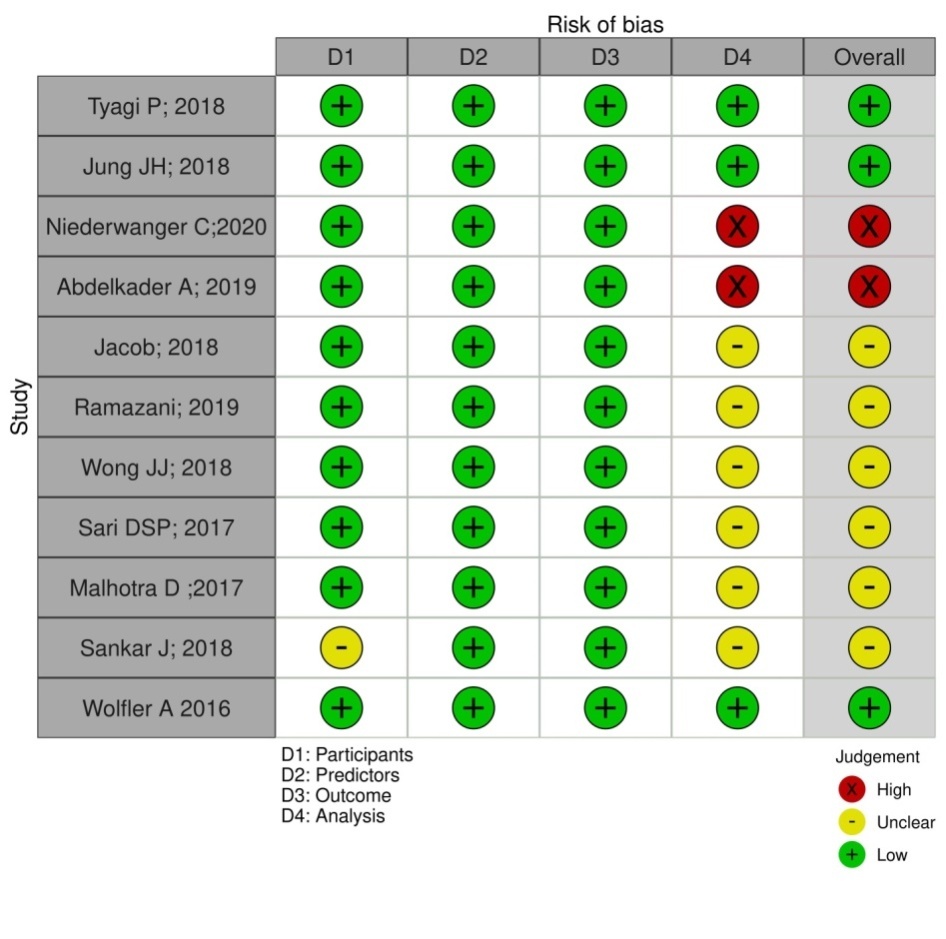


Supplementary Figure 5B: Risk of Bias assessment using PROBAST tool showing overall risk of bias

**
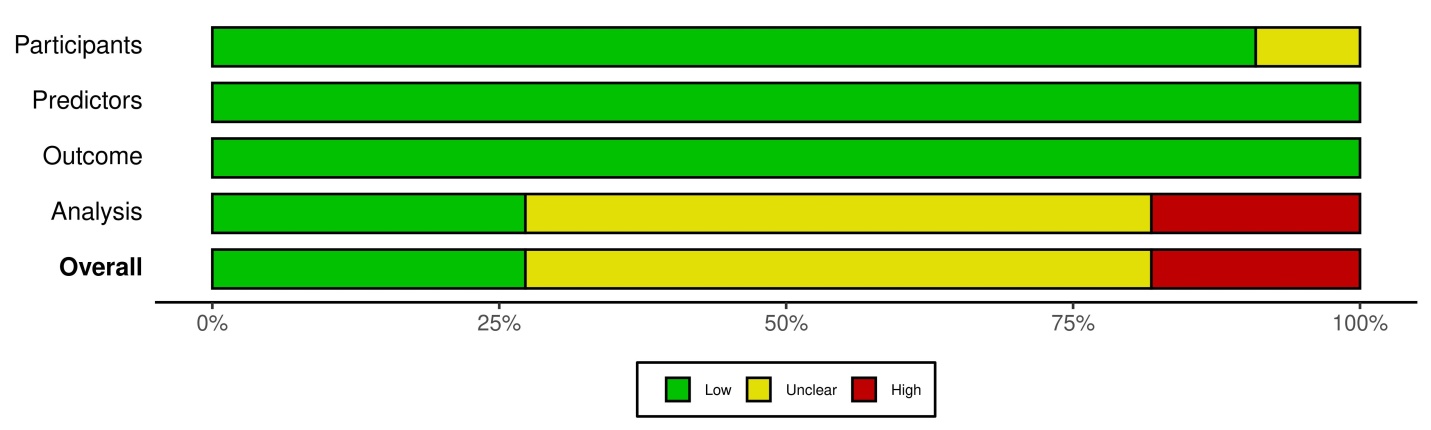
**

**Supplementary Figure 6 : Meta-regression analysis to explore the source of heterogeneity for PIM-3 scoring system**


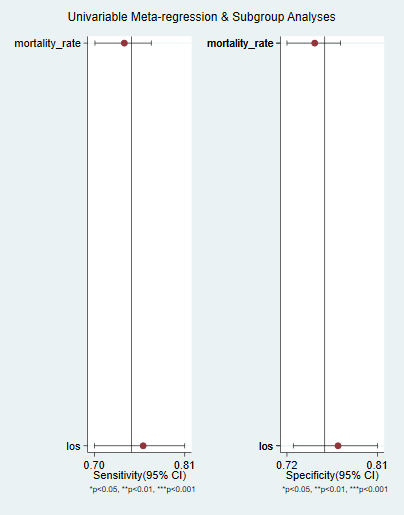


Supplementary figure 7: Publication bias in PELOD-2 studies


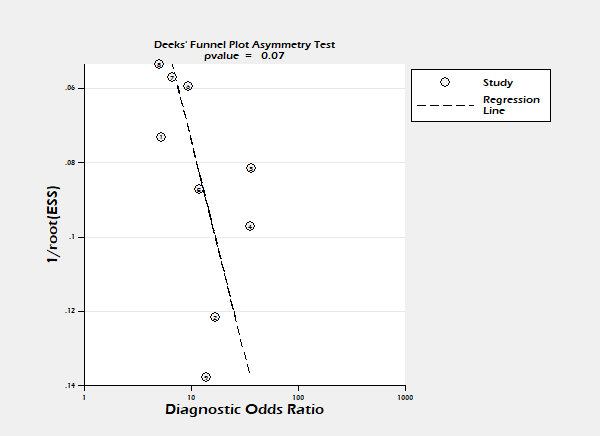


Supplementary figure 8A: Methodological quality for PELOD-2 studies using PROBAST tool


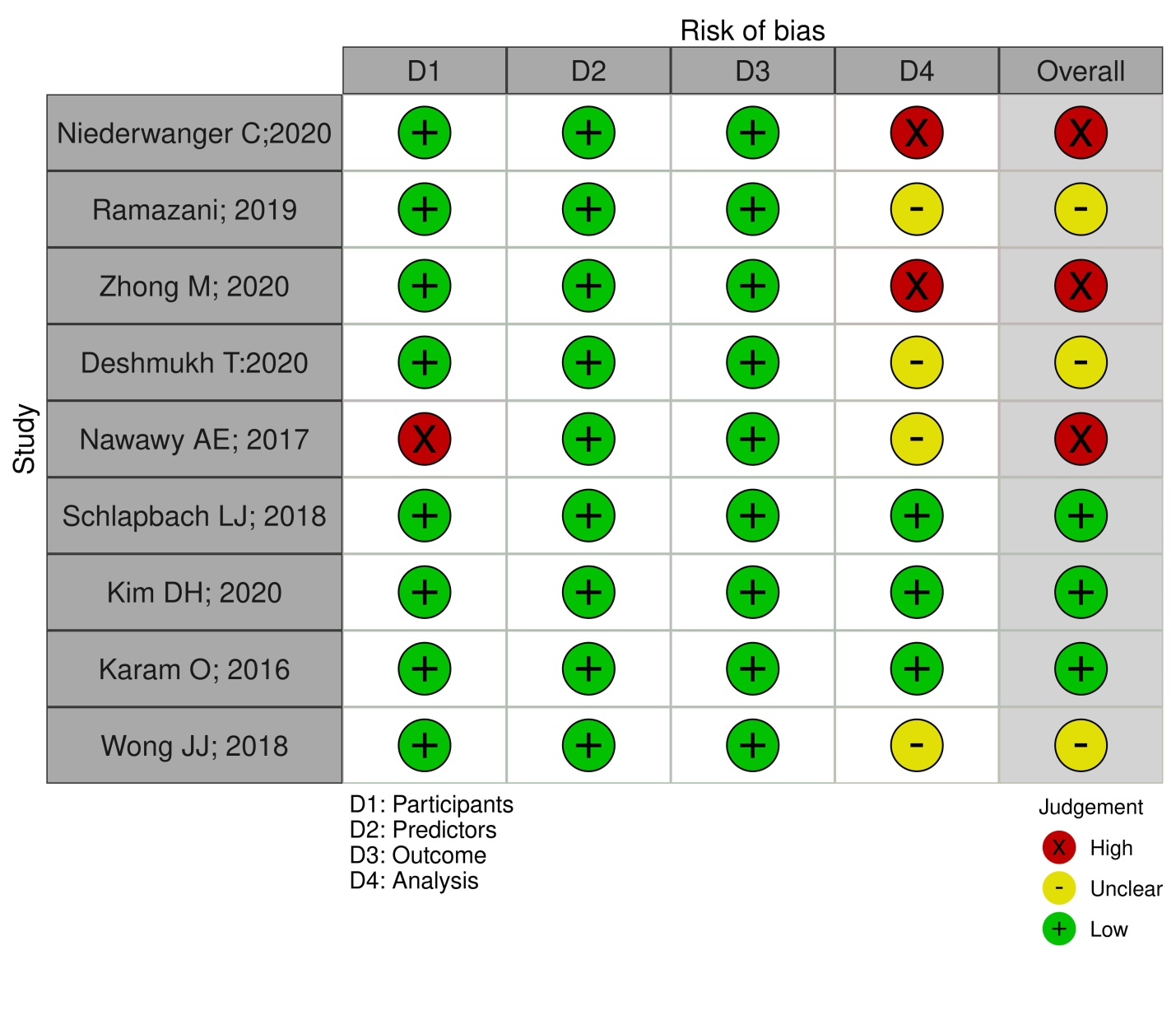


Supplementary figure 8B: Methodological quality for PELOD-2 studies using PROBAST pool


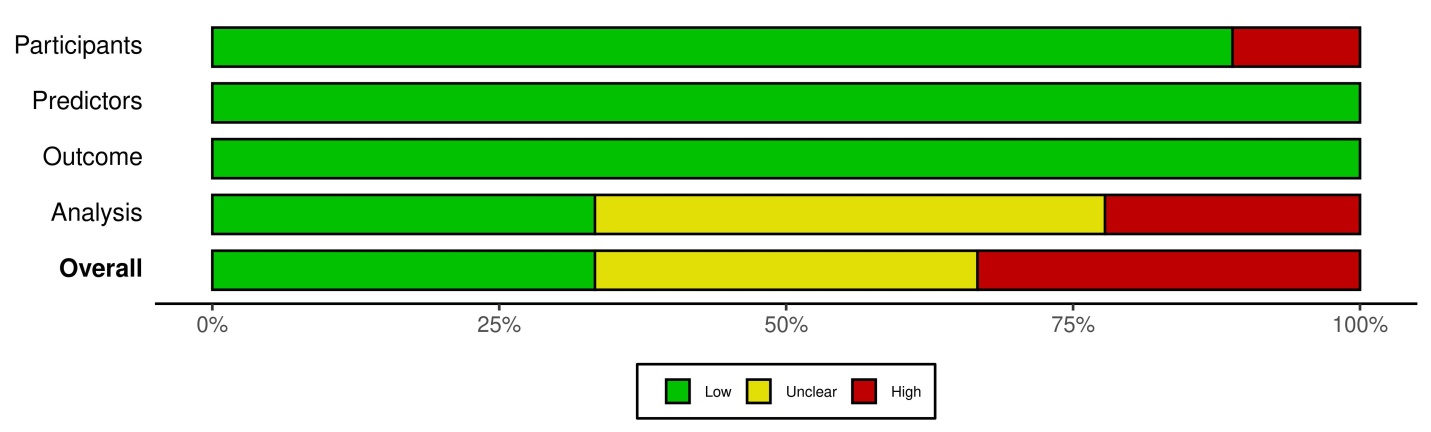


Supplementary Figure 9 : Meta-regression analysis for determining the influential factor for pooled effect size for PELOD-2 studies

**
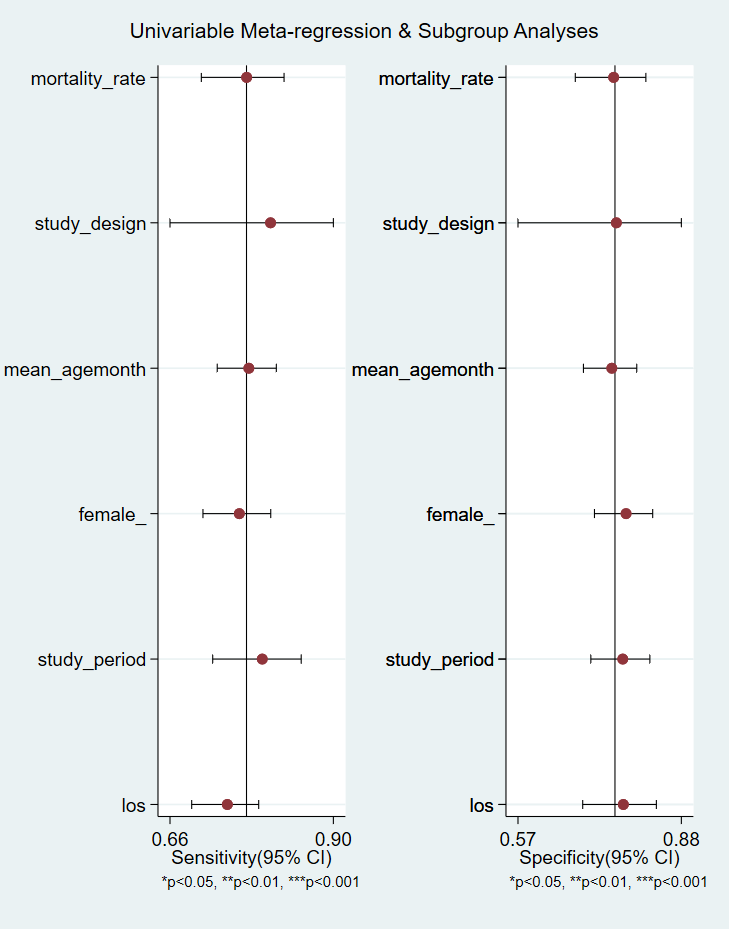
**

**Supplementary table 1 : List of excluded studies with reasons**

| **Study no. / Score** | **Author** | **Year** | **Reason for exclusion** | **Key finding** |
| --- | --- | --- | --- | --- |
| **Prism III/IV** |  |  |  |  |
|  | Zhang Z (III/IV) | 2020 | No dataset was available regarding sensitivity & specificity | Performance of PCIS was inferior to PRISM IV or PELOD-2 |
|  | Goncalves J P (III) | 2015 | No dataset was available regarding sensitivity & specificity | PRISM-III had good discrimination.  PELOD-2 needs recalibration to be a better reliable prediction tool. |
|  | Horvat CM | 2019 | No dataset was available regarding sensitivity & specificity | Electronically derived intensive care acuity scores demonstrate very good to excellent discrimination and can be calibrated to institutional outcomes. This approach can facilitate both performance improvement and research initiatives and may offer a scalable strategy for comparison of interinstitutional PICU outcome |
|  | Rr P (III) |  | No dataset was available regarding sensitivity & specificity | Emergency respiratory and neurology admissions and previous bacteremia were independent risk factors for 60-day mortality for pediatric oncological patients admitted to the PICU. |
|  | Pollack MM | 1996 | Relevant data could not extracted | PRISM-III has excellent prognostic significance in PICU patients |
| **PELOD-2** |  |  |  |  |
|  | Leteurtre S | 2015 | No dataset was available regarding sensitivity & specificity | This study suggests that the progression of the severity of organ dysfunctions can be evaluated by measuring the dPELOD-2 score during a set of 7 days in PICU, providing useful information on outcome in critically ill children. Its external validation would be useful. |
|  | Leclerc F | 2014 | No dataset was available regarding sensitivity & specificity | study demonstrates that the non- respiratory Paediatric Logistic Organ Dysfunction-2 score of the ntire PICU stay is highly predictive of death in children with acute respiratory failure of whom 94.3% were invasively ventilated. The non-respiratory Paediatric Logistic Organ Dysfunction-2 score could represent the non- respiratory organ failure definition tool whose development was recommended in the international expert recommendations on paediatric acute respiratory distress syndrome |
| **PIM 3** |  |  |  |  |
|  | Lee OK | 2016 | No dataset was available regarding sensitivity & specificity | the performance of the PIM3 scoring system in Korean patients aged < 18 years was good |
|  | Straney L | 2013 | No dataset was available regarding sensitivity & specificity | scoring system has good prognostic significance in PICU patients. |

**Supplementary Table 2: Question**: Should [PRISM III/IV] be used to predict [mortality] in [PICU patients]?

| \| Sensitivity \| 0.78 (95% CI: 0.72 to 0.83) \| \| --- \| --- \| \| Specificity \| 0.75 (95% CI: 0.68 to 0.81) \| |  | \| Prevalences \| 15% \| 20% \| 25% \| \| --- \| --- \| --- \| --- \| |  |
| --- | --- | --- | --- | --- | --- | --- | --- | --- | --- | --- | --- |

| Outcome | № of studies (№ of patients) | Study design | Factors that may decrease certainty of evidence | | | | | Effect per 1,000 patients tested | | | Test accuracy CoE |
| --- | --- | --- | --- | --- | --- | --- | --- | --- | --- | --- | --- |
|  |  |  | Risk of bias | Indirectness | Inconsistency | Imprecision | Publication bias | pre-test probability of 15% | pre-test probability of 20% | pre-test probability of 25% |  |
| **True positives** (patients with [target condition]) | 19 studies 1367 patients | cross-sectional (cohort type accuracy study) | serious | not serious | very serious | not serious | none | 117 (108 to 124) | 156 (144 to 166) | 195 (180 to 208) | ⨁◯◯◯ VERY LOW |
| **False negatives** (patients incorrectly classified as not having [target condition]) |  |  |  |  |  |  |  | 33 (26 to 42) | 44 (34 to 56) | 55 (42 to 70) |  |
| **True negatives** (patients without [target condition]) | 19 studies 8201 patients | cross-sectional (cohort type accuracy study) | serious | not serious | very serious | not serious | none | 638 (578 to 689) | 600 (544 to 648) | 563 (510 to 608) | ⨁◯◯◯ VERY LOW |
| **False positives** (patients incorrectly classified as having [target condition]) |  |  |  |  |  |  |  | 212 (161 to 272) | 200 (152 to 256) | 187 (142 to 240) |  |

**Supplementary Table 3 : Question**: Should [PIM-3] be used to diagnose [mortality] in [PICU]?

| \| Sensitivity \| 0.75 (95% CI: 0.71 to 0.79) \| \| --- \| --- \| \| Specificity \| 0.76 (95% CI: 0.73 to 0.79) \| |  | \| Prevalences \| 15% \| 20% \| 25% \| \| --- \| --- \| --- \| --- \| |  |
| --- | --- | --- | --- | --- | --- | --- | --- | --- | --- | --- | --- |

| Outcome | № of studies (№ of patients) | Study design | Factors that may decrease certainty of evidence | | | | | Effect per 1,000 patients tested | | | Test accuracy CoE |
| --- | --- | --- | --- | --- | --- | --- | --- | --- | --- | --- | --- |
|  |  |  | Risk of bias | Indirectness | Inconsistency | Imprecision | Publication bias | pre-test probability of 15% | pre-test probability of 20% | pre-test probability of 25% |  |
| **True positives** (patients with [mortality]) | 11 studies 998 patients | cross-sectional (cohort type accuracy study) | serious | not serious | not serious | not serious | none | 112 (107 to 119) | 150 (142 to 158) | 188 (178 to 198) | ⨁⨁⨁◯ MODERATE |
| **False negatives** (patients incorrectly classified as not having [mortality]) |  |  |  |  |  |  |  | 38 (31 to 43) | 50 (42 to 58) | 62 (52 to 72) |  |
| **True negatives** (patients without [mortality]) | 11 studies 14804 patients | cross-sectional (cohort type accuracy study) | serious | not serious | very serious | not serious | none | 646 (620 to 672) | 608 (584 to 632) | 570 (548 to 593) | ⨁◯◯◯ VERY LOW |
| **False positives** (patients incorrectly classified as having [mortality]) |  |  |  |  |  |  |  | 204 (178 to 230) | 192 (168 to 216) | 180 (157 to 202) |  |

**Supplementary table 4 : Question**: Should [PELOD-2] be used to diagnose [mortality] in [PICU]?

| \| Sensitivity \| 0.78 (95% CI: 0.71 to 0.83) \| \| --- \| --- \| \| Specificity \| 0.75 (95% CI: 0.68 to 0.81) \| |  | \| Prevalences \| 15% \| 20% \| 25% \| \| --- \| --- \| --- \| --- \| |  |
| --- | --- | --- | --- | --- | --- | --- | --- | --- | --- | --- | --- |

| Outcome | № of studies (№ of patients) | Study design | Factors that may decrease certainty of evidence | | | | | Effect per 1,000 patients tested | | | Test accuracy CoE |
| --- | --- | --- | --- | --- | --- | --- | --- | --- | --- | --- | --- |
|  |  |  | Risk of bias | Indirectness | Inconsistency | Imprecision | Publication bias | pre-test probability of 15% | pre-test probability of 20% | pre-test probability of 25% |  |
| **True positives** (patients with [mortality]) | 9 studies 500 patients | cross-sectional (cohort type accuracy study) | serious | not serious | serious | not serious | none | 117 (107 to 124) | 156 (142 to 166) | 195 (178 to 208) | ⨁⨁◯◯ LOW |
| **False negatives** (patients incorrectly classified as not having [mortality]) |  |  |  |  |  |  |  | 33 (26 to 43) | 44 (34 to 58) | 55 (42 to 72) |  |
| **True negatives** (patients without [mortality]) | 9 studies 3180 patients | cross-sectional (cohort type accuracy study) | serious | not serious | very serious | not serious | none | 638 (578 to 689) | 600 (544 to 648) | 563 (510 to 608) | ⨁◯◯◯ VERY LOW |
| **False positives** (patients incorrectly classified as having [mortality]) |  |  |  |  |  |  |  | 212 (161 to 272) | 200 (152 to 256) | 187 (142 to 240) |  |
